# Organic Salts of *p*-Coumaric Acid and *Trans*-Ferulic Acid with Aminopicolines

**DOI:** 10.3390/molecules25030751

**Published:** 2020-02-10

**Authors:** Sosthene Nyomba Kamanda, Ayesha Jacobs

**Affiliations:** Chemistry Department, Faculty of Applied Sciences, Cape Peninsula University of Technology, PO Box 1906, Bellville 7535, South Africa; sosthenekam@gmail.com

**Keywords:** p-coumaric acid, trans-ferulic acid, organic sals, hydroxycinnamic acids, multicomponent crystals

## Abstract

*p*-Coumaric acid (*p*CA) and *trans*-ferulic acid (TFA) were co-crystallised with 2-amino-4-picoline (2A4MP) and 2-amino-6-picoline (2A6MP) producing organic salts of (*p*CA^−^)(2A4MP^+^) (**1**), (*p*CA^–^ )(2A6MP^+^) (**2**) and (TFA^–^ )(2A4MP^+^)·($\frac{3}{2}$H_2_O) (**3**). For salt **3**, water was included in the crystal structure fulfilling a bridging role. *p*CA formed a 1:1 salt with 2A4MP (*Z*’ = 1) and a 4:4 salt with 2A6MP (*Z*’ = 4). The thermal stability of the salts was determined using differential scanning calorimetry (DSC). Salt **2** had the highest thermal stability followed by salt **1** and salt **3**. The salts were also characterised using Fourier transform infrared (FTIR) spectroscopy. Hirshfeld surface analysis was used to study the different intermolecular interactions in the three salts. Solvent-assisted grinding was also investigated in attempts to reproduce the salts.

## 1. Introduction

Multicomponent crystals are structurally homogeneous crystalline materials containing two or more building blocks present in definite stoichiometric amounts [1,2,3]. The design and synthesis of multicomponent crystals have considerable therapeutic and commercial benefits as they often lead to improvement of physicochemical properties like solubility, bioavailability and stability [4,5]. Multicomponent crystals encompass co-crystals, organic salts, hydrates and solvates.

Organic salts are multicomponent crystals that can be identified by the transfer of a proton between an acid and a base [6]. An organic salt can improve the physicochemical properties such as solubility of an API 100–1000 times more than co-crystals. Thus, 50% of the drugs on the market are in a salt form [7]. The most studied donor group is the carboxylic acid [8]. The reaction of an acid and a base will produce organic salts or co-crystals depending on the p*K*_a_ difference between the acid and the base. According to the previous observations on the p*K*_a_ rule in the literature, if the Δp*K*_a_ (p*K*_a_ base − p*K*_a_ acid) is less than −1, non-ionised acid–base complexes are exclusively observed. Ionised acid–base complexes are exclusively expected for Δp*K*_a_ ≥ 4 and for Δp*K*_a_ between −1 and 4, there is a probability of either salt or co-crystal formation [9,10,11].

**Scheme 1:**
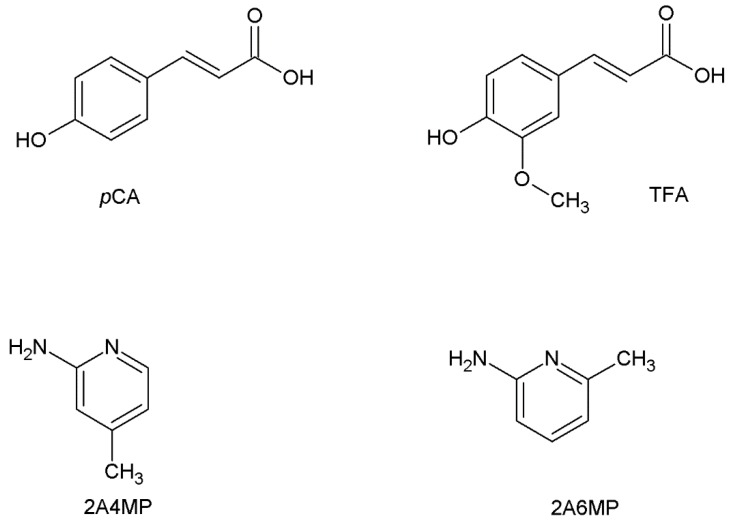
Chemical structures of *p*-coumaric acid (*p*CA), *trans*-ferulic acid (TFA), 2-amino-4-picoline (2A4MP) and 2-amino-6-picoline (2A6MP).

This study focuses on the formation of organic salts of *p*-coumaric acid (*p*CA) and *trans*-ferulic acid (TFA), both derivatives of hydroxycinnamic acid. They are classified as phytochemical and nutraceutical molecules and are found in various plant species, such as peanuts, carrots and tomatoes [12,13]. They possess antioxidant and anti-inflammatory properties [14,15,16]. *p*CA and TFA are the most prevalent hydroxycinnamic acids [17]. They differ structurally due to the substituted methoxy group present in the *meta* position in TFA. *p*CA and TFA both contain carboxylic acid (strong hydrogen bond donor) and hydroxyl (hydrogen bond donor and/or acceptor) functional groups. These functional groups compete in the formation of supramolecular synthons in crystalline solids [18]. A CSD search (version 5.40, August 2019 update) [19] revealed only five multicomponent crystal structures of *p*-coumaric acid (*p*CA) and seven of *trans*-ferulic acid (TFA). In this study, 2-amino-4-picoline (2A4MP) and 2-amino-6-picoline (2A6MP) were used as co-crystal formers (Scheme 1).

## 2. Results and Discussion

The Δp*K*_a_ for *p*CA with 2A4MP is 3.61; for *p*CA with 2A6MP Δp*K*_a_ = 3.59, whereas Δp*K*_a_ for TFA with 2A4MP is 4.04 [20]. Thus, a higher probability of salt formation is expected for these compounds as Δp*K*_a_ is between 3 and 4 [9]. No crystals were obtained for TFA with 2A6MP.

**Figure 1:**
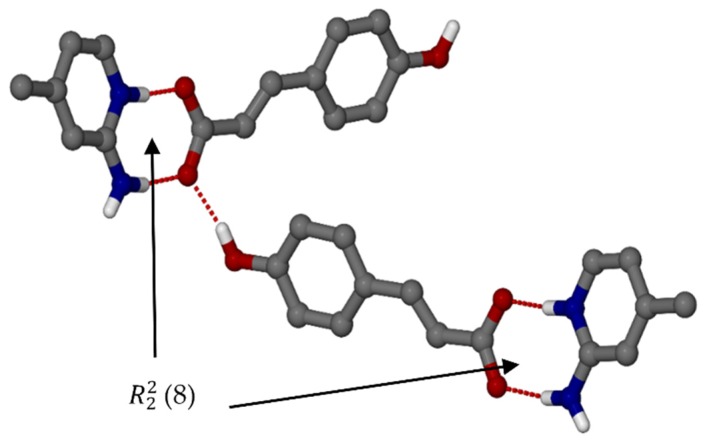
Hydrogen bonding with motif ring of organic salt **1**.

**Figure 2:**
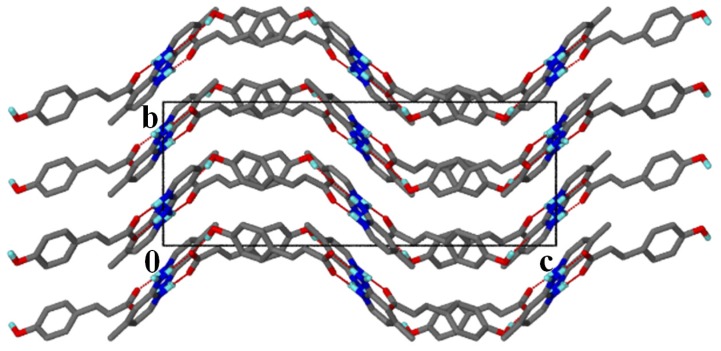
Packing diagram of salt **1** along [100].

Organic salt **1**, (*p*CA^−^)(2A4MP^+^), crystallised in the orthorhombic space group *Pbca* with *Z* = 8. The deprotonated carboxylic acid group of *p*CA is linked to both nitrogen atoms of 2A4MP *via* N-H···O interactions forming $R_{2}^{2}$ (8) rings; it is also hydrogen bonded to the phenolic OH of another *p*CA^−^ anion resulting in $C_{1}^{1}\left(10\right)$ chains [21]. The 2-aminopyridinium carboxylate heterosynthon is known to be a robust interaction [22], see Figure 1. The second amine hydrogen atom is involved in a weaker N-H···O interaction with the carboxylate group of a neighbouring *p*CA^−^ anion forming $C_{2}^{2}\left(6\right)$ chains. These interactions result in a 2D network along the *c* axis (Figure 2).

**Figure 3:**
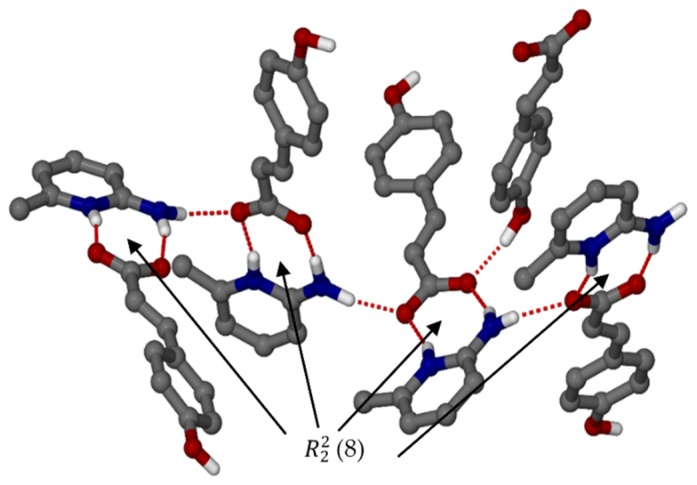
Hydrogen bonding with motif ring of organic salt **2**.

Organic salt **2**, (*p*CA^−^)(2A6MP^+^), crystallised in the monoclinic space group *P*2_1_ with Z = 8. Again, the 2-aminopyridinium carboxylate heterosynthon forms $R_{2}^{2}$ (8) rings with a $C_{2}^{2}\left(20\right)$ chain which is a result of alternating *p*CA^−^ and 2A6MP^+^ ions (Figure 3). These interactions form a complex 3D network. It is interesting to note that this salt has Z’ = 4 unlike the previous structure which had *Z*’= 1. The density of this salt is lower than that for salt **1**, thus less close packing is possible when the methyl group changes position in 2A4MP (*para* to the pyridine nitrogen) vs 2A6MP (*ortho* to the pyridine nitrogen). Salt **2** also displays moderate hydrogen bonds in the range 2.606(3) to 2.941(3) Å [23], compared to salt **1**, which has three moderate (2.662(2)–2.893(3) Å) and one weaker hydrogen bond of 3.365(2) Å. Thus, the lower density for salt **2** is accompanied by shorter hydrogen bonds compared to salt **1**, and this is typical for high *Z*’ structures [24,25,26].

**Figure 4:**
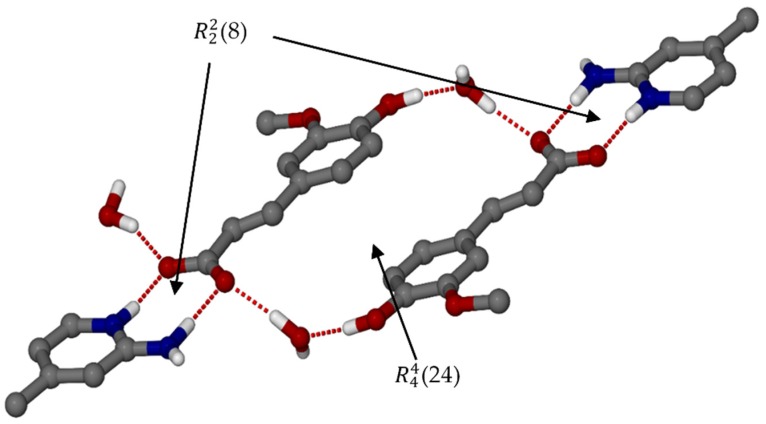
Hydrogen bonding with the ring motif of organic salt **3**.

Organic salt **3**, (TFA^−^)(2A4MP^+^) ($\frac{3}{2}$H_2_O), was successfully solved in the monoclinic space group C2/*c* and again the 2-aminopyridinium carboxylate heterosynthon is observed forming $R_{2}^{2}$ (8) rings. The TFA^−^ anions are bridged by water molecules resulting in $R_{4}^{4}$ (24) rings and $C_{2}^{2}\left(14\right)$ chains. The TFA^−^ anions are disordered over two positions with the site occupancy of the major component, 0.80837. The hydrogen bonding is shown in Figure 4 with the major component of the TFA^−^ anion shown. Both *p*CA and TFA contain hydroxyl and carboxylic acid groups which compete in synthon formation [18]. For all three structures, the supramolecular heterosynthons were preferred over the homosynthons with the persistence of the 2-aminopyridinium carboxylate heterosynthon. In addition, this synthon was favoured in the presence of the competing hydroxyl group. Furthermore the hydroxyl group participated in this interaction by acting as a hydrogen bond donor to the carboxylate forming a three-centred (bifurcated) hydrogen bond. For salt **3**, the hydroxyl group interacts with the carboxylate via the water molecule. TFA also has a methoxy group, which reduces the donating ability of the phenolic OH group compared to the carboxylic acid [27], and did not affect the formation of the 2-aminopyridinium carboxylate heterosynthon. In a recent article, the crystal structures of two aminopyridinium citrate salts were reported, the 3:1 2-aminopyridinium:citrate salt displayed the 2-aminopyridinium carboxylate heterosynthon whereas the other 1:1 salt did not [28].

**Figure 5:**
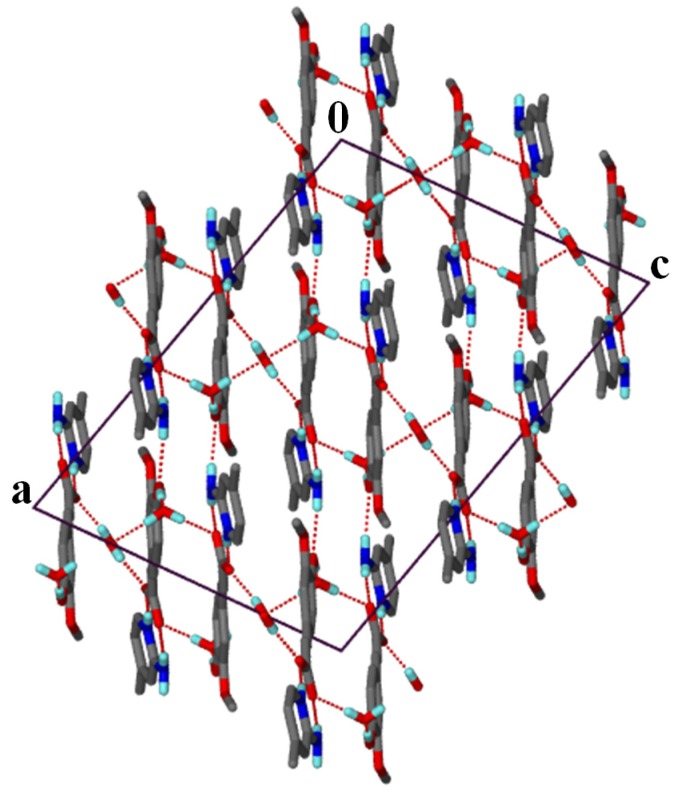
Packing diagram of salt **3** along [010].

Note that neighbouring TFA^−^ anions are oriented parallel to each other with the distance between the ring centroids approximately 3.856 Å. This could be due to the steric hindrance of the methoxy group on the aromatic ring, as for salt **1** the adjacent *p*CA^−^ anions are nearly orthogonal. In salt **3**, the water molecules act as a space filler and play a bridging role linking the hydroxyl group to the carboxylate of neighbouring TFA^−^ anions. The $R_{4}^{4}\;$(24) rings form columns that are linked by water molecules (Figure 5). The water molecule located on a twofold axis and sandwiched between the columns is hydrogen bonded to two TFA^−^ anions and two other water molecules.

### 2.1. PXRD Analysis

**Figure 6:**
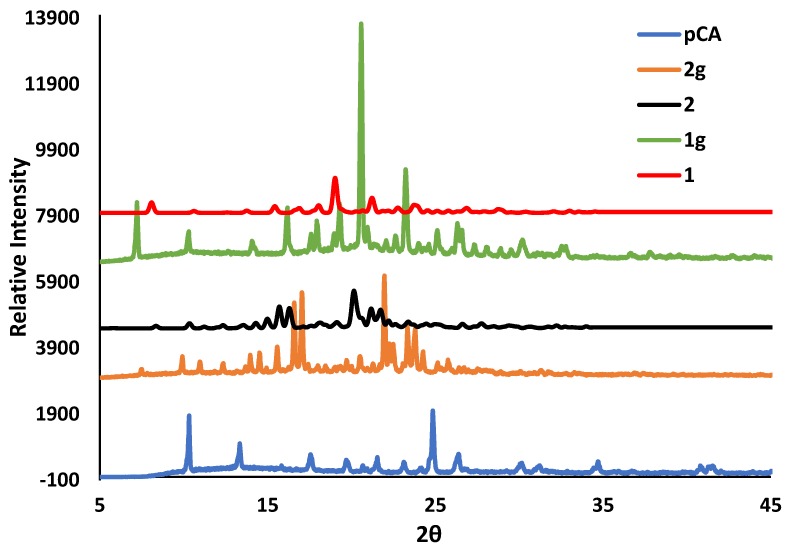
PXRD patterns for *p*CA, the ground products **1g** and **2g** after 30 min and the calculated organic salts **1** and **2**.

**Figure 7:**
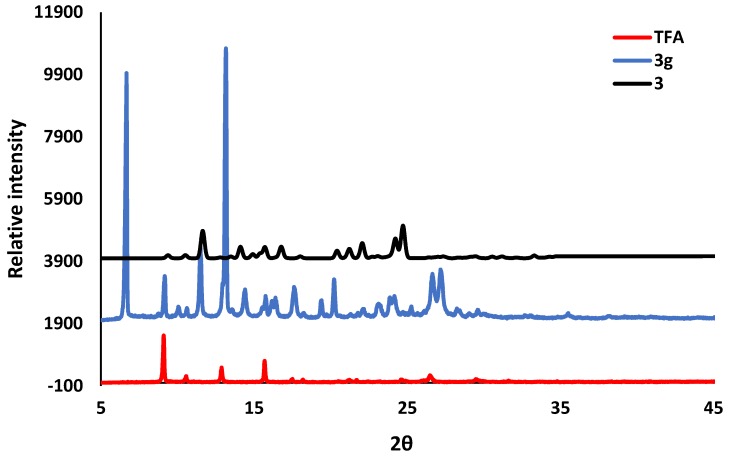
PXRD pattern for TFA compared with the ground product **3g** after 20 min and the calculated organic salt **3**.

**Figure 8:**
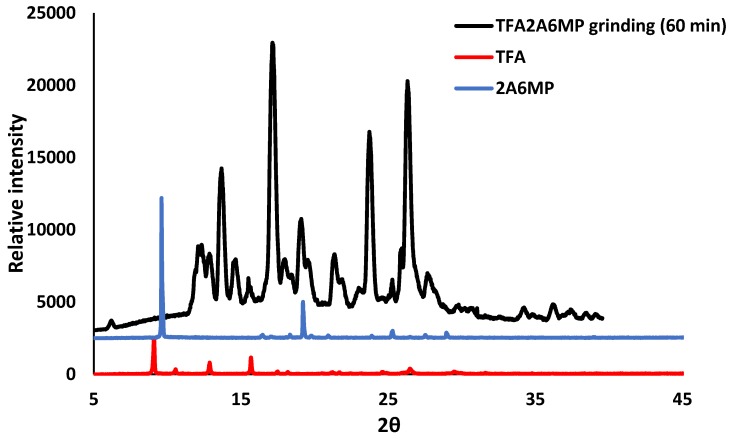
PXRD analyses of the ground product of TFA and 2A6MP after 60 min (black), TFA (red) and 2A6MP (blue).

Solvent-assisted grinding was used in an attempt to reproduce the organic salts, and an equimolar mixture of the acid and the co-former were placed in a mortar and ground for 20 to 30 min with a few drops of the solvent mixture, 75:25 (*v/v*) ethanol and 1,2-dichloroethane. For the grinding experiment of TFA and 2A4MP, a few drops of water were also added. The PXRD patterns of the ground products **1g**, **2g** and **3g** were compared with their respective starting materials and the calculated patterns of organic salts **1**, **2** and **3** from LAZY PULVERIX [29]. For *p*CA and 2A4MP, partial reaction occurred (**1g**, Figure 6). For *p*CA and 2A6MP partial reaction was also observed but unidentified peaks were also found in the PXRD pattern of the ground product (**2g**). This grinding experiment was repeated for 60 min and the PXRD pattern showed a better match to that of the calculated one; however, there were still unidentified peaks, thus confirming the presence of a mixture. The corresponding PXRD patterns have been deposited in the supplementary data. The ground product **3g** obtained after 20 min grinding appears to be a mixture of starting material, salt **3** and an unidentified product (Figure 7). Thus, the solvent-assisted grinding experiments were partially successful in the preparation of the salts. The grinding experiment of TFA and 2A6MP was also attempted even though no single crystals were obtained. A 1:1 mixture of TFA and 2A6MP was ground for 60 min using the same solvent mixture used in the previous experiments. The PXRD pattern of the resultant powder showed new peaks when compared to those of the starting materials, indicating that a new compound was formed (Figure 8). There were also residual peaks of 2A6MP present, thus the reaction was incomplete. Mechanochemical syntheses is a popular technique for preliminary co-crystallisation experiments as it gives quick results and requires either no solvent or a minimum amount of solvent [30]. The grinding experiments of these hydroxycinnamic acids gave promising results even though partial reaction occurred, possibly due to the manual grinding technique which requires longer reaction times than an automatic device. However, these preliminary experiments could pave the way for further study into the structural landscape of *p*CA and TFA which is underexplored as shown by the few crystal structures present in the CSD.

### 2.2. Infrared Spectroscopy

The absence of the carboxylic acid OH stretching broad band of *p*CA around the 3000 cm^−1^ region was observed for both salts **1** and **2**, confirming the proton transfer between the starting material and the co-formers. Salt **1** shows two small peaks around those regions corresponding to the primary amine (NH_2_) of 2A4MP. For salt **2**, no primary amine peak is shown but a shifted peak was observed, which is assigned to the hydrogen bonding between the phenolic OH of *p*CA and the carbonyl oxygen of another *p*CA in the crystal structure. Similarly, salt **3** does not show a carboxylic OH stretching band but a shift due to the hydrogen bonding between the phenolic OH of TFA and the water molecule, is observed at approximately 3000 to 3700 cm^−1^. The FTIR spectra of the salts are given in the supplementary data.

### 2.3. Thermal Analysis

**Figure 9:**
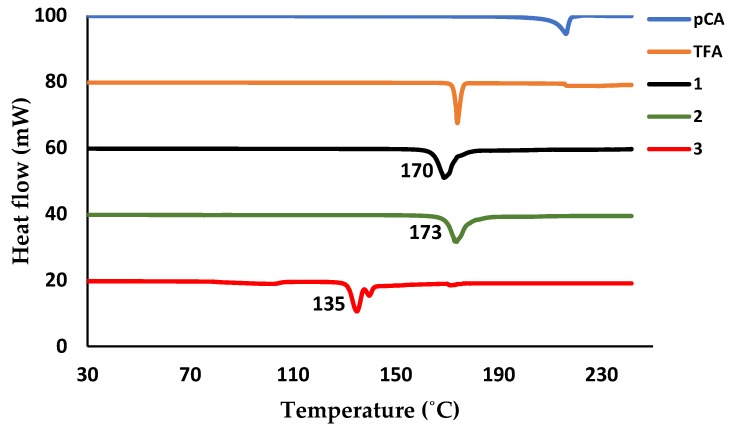
Differential scanning calorimetry (DSC) plot of *p*CA (blue), TFA (brown), salt **1** (black), salt **2** (green) and salt **3** (red).

The differential scanning calorimetry (DSC) curves of the organic salts displayed one endothermic peak which corresponds to the melting points of the compounds. All the salts have melting peaks occurring at temperatures in between those of the starting materials, Figure 9. The melting point of salt **1** results in an endothermic peak at 170 °C, which differs from the melting points of *p*CA (214.9 °C) and 2A4MP (100 °C). The endothermic peak for salt **2** occurs at a slightly higher temperature (173 °C) compared to salt **1** (T_peak_ of 2A6MP = 44.9 °C). Salt **3** has the lowest melting point at 135 °C (T_peak_ for TFA =174 °C), the inclusion of water could have contributed to the lower melting point of this salt.

### 2.4. Torsion Angles

**Figure 10:**
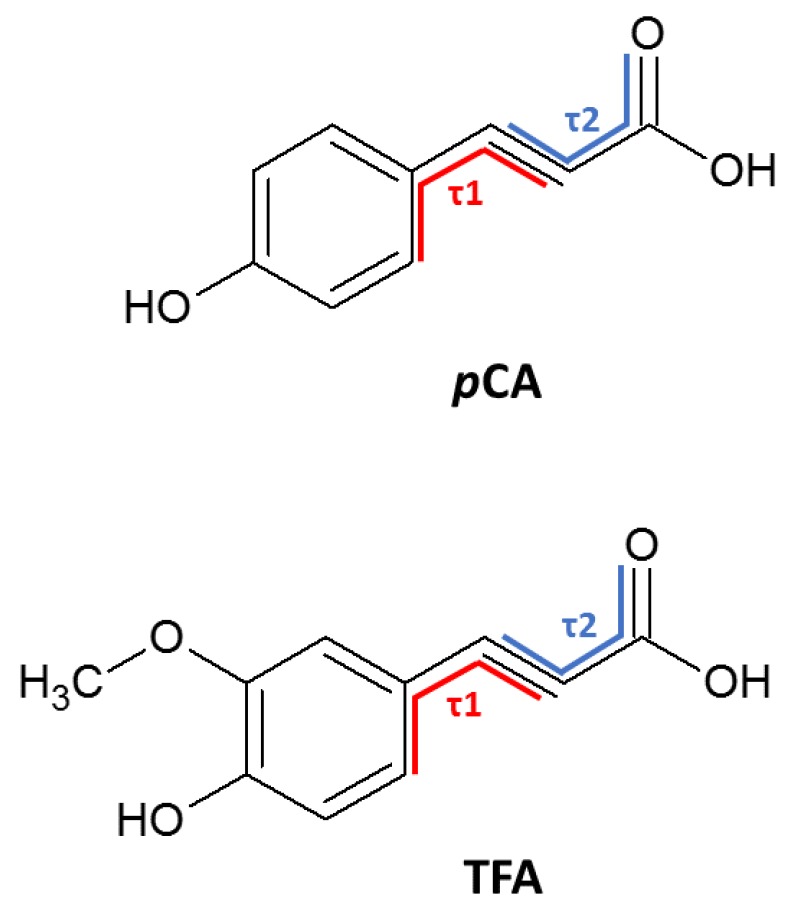
Torsion angles of *p*CA and TFA.

Figure 10 depicts the torsion angles considered in this study for both *p*CA and TFA. The torsion angles for all four *p*CA^−^ anions in salt **2** were obtained and significant differences were found. For this salt, $\tau_{1}$ varied from 0.5 to 14.4° and $\tau_{2}$ varied from 5.1 to 26.7°. Salt **1** showed similar flexibility in the rotation of the ring ($\tau_{1}$) compared to salt **2** with $\tau_{1}$ = 12.8° and $\tau_{2}$ = 10.1°. TFA in salt **3** has both the lowest ring twist torsion angle ($\tau_{1}$ = 0.2°) and the lowest carboxylic acid twist torsion angle ($\tau_{2}$ = 4.9°).

### 2.5. Hirshfeld Surface Analysis

For all three salts, the leading interactions are as follows, H···H > O···H > C···H. The π···π stacking represented by C···C has a lower contribution to the stability of the salts, with salt **3** displaying the highest value of 7.5%. Similar percentage contributions were found for the O···H interactions in the three salts (average 30.8%). For all three salts, the C···H contacts are shown as “chicken wings”. The H···H contacts (average 40.1%) are prominent in the molecular packing and appear as scattered points in the middle region of the 2D fingerprint plots [31].

## 3. Materials and Methods

All chemicals were obtained from Sigma Aldrich (Schnelldorf, Germany) and were used without further purification.

### 3.1. Crystallisation

The salts were obtained by dissolving the target compound and co-former (1:1 molar ratio) in a solvent mixture followed by gentle heating on a hot plate and stirring until the solution became clear. The solutions were left at room temperature until crystallisation occurred. For (*p*CA^−^)(2A4MP^+^) (**1**) and (*p*CA^−^)(2A6MP^+^) (**2**) a 75:25 (v/v) ethanol and 1,2-dichloroethane solvent mixture was used and crystals were obtained after 1 week. For (TFA^−^)(2A4MP^+^)·($\frac{3}{2}$H_2_O) (**3**) a similar solvent mixture was used; however, a few drops of water were added to give a clear solution. Slow evaporation of this solution gave crystals after 4 days.

### 3.2. Thermal Analysis

Differential scanning calorimetry (DSC) was conducted on a Perkin–Elmer 6000 (PerkinElmer Inc., Waltham, MA, USA) with a nitrogen gas purge at 20 mL min^−1^. Experiments were performed from 30 to 250 °C at a heating rate of 10 °C min^−1^.

### 3.3. Infrared Spectroscopy

IR spectra were measured on a PerkinElmer Spectrum Two FTIR spectrometer (PerkinElmer Inc., Waltham, MA, USA) equipped with an ATR Diamond accessory for powder samples. Samples were scanned over a range of 400 to 4000 cm^−1^.

### 3.4. Powder X-ray Diffraction

The powder X-ray diffraction patterns of the samples were recorded with a D2 Phaser Bruker diffractometer (Bruker, Karlsruhe, Germany) with Cu-K*α* radiation of 1.54184 Å. The samples were scanned between 4 and 50° 2*θ* and the voltage tube and amperage were at 30 kV and 10 mA max, respectively, with an Xflash detector and a scintillation counter, 1-dim LYNXEYE.

### 3.5. Crystal Structure Determination

Single crystal X-ray diffraction data were recorded on a Bruker KAPPA APEX II DUO diffractometer (Bruker, Karlsruhe, Germany) using graphite monochromated Mo-K*α* radiation (*λ* = 0.71073 Å) at 173 K. Data were corrected for Lorentz-polarization effects and for absorption (SADABS) [32]. The structures were solved by direct methods in SHELXS and refined by full-matrix least-squares on *F*^2^ using SHELXL [33] within the interface XSEED [34]. The non-hydrogen atoms were found in the difference electron density map and were refined anisotropically, whereas hydrogen atoms were placed in calculated positions and refined with isotropic temperature factors. Details of the crystal structure refinements are given in Table 1.

## 4. Conclusions

The p*K*_a_ rule predicted that both *p*CA and TFA would form salts with the aminopicolines. The salts (*p*CA^−^)(2A4MP^+^), (*p*CA^−^)(2A6MP^+^) and (TFA^−^)(2A4MP^+^)·($\frac{3}{2}$H_2_O) were analysed using single crystal X-ray diffraction. No suitable crystals were obtained for TFA and 2A6MP however grinding experiments revealed that a new compound was formed. The aminopyridinium carboxylate supramolecular heterosynthon was favoured in the presence of the hydroxyl group in the case of *p*CA, and it was also preferred in the presence of both hydroxyl and methoxy groups for TFA. For the *p*CA salts, the phenol OH hydrogen bonds to the carboxylate and in the case of the TFA salt, water forms a bridge between the phenol OH and the carboxyl group. *p*CA gave a 1:1 salt with 2A4MP and a 4:4 salt with 2A6MP. The change in position of the methyl group in the aminopicolines influenced the packing. Similarly the presence of the methoxy group in TFA compared to *p*CA also resulted in a different packing arrangement for the salts involving 2A4MP. The thermal stability trend as determined by the melting points for the salts is **2** > **1** > **3**.

## Figures and Tables

**Table 1:** Crystallographic data for salts **1**, **2** and **3**.

Organic Salts	1	2	3
Molecular formula	C_15_H_16_N_2_O_3_	C_60_H_64_N_8_O_12_	C_16_H_21_N_2_O_6_
*M_r_* (g/mol)	272.30	1089.19	329.35
Temperature (K)	173	173	173
Crystal system	Orthorhombic	Monoclinic	Monoclinic
Space group	*Pbca*	*P*2_1_	C2/*c*
*a* (Å)	12.056(2)	13.930(3)	20.111(4)
*b* (Å)	9.1511(18)	9.948(2)	12.016(2)
*c* (Å)	25.048(5)	20.797(4)	14.226(3)
*α* (°)	90	90	90
*β* (°)	90	94.33(3)	104.96(3)
*γ* (°)	90	90	90
*V* (Å^3^)	2763.4(9)	2873.6(10)	3321.4(12)
*Z*	8	2	8
*λ* (Å)	0.71073	0.71073	0.71073
*ρ* (calcd) (g/cm^3^)	1.309	1.259	1.317
Absorption coefficient *μ* (mm^−1^)	0.092	0.089	0.100
2*θ*_max_ (°)	56.6	55.8	56.7
Reflections collected	26738	23267	24936
No. data with *I* > 2*σ*(*I*)	3424	13608	4149
No. parameters	198	789	288
Final *R* (*I* > 2*σ*(*I*))	*R*_1_ = 0.0460; *wR*_2_ = 0.1049	*R*_1_ = 0.0574; *wR*_2_ = 0.1372	*R*_1_ = 0.0431; *wR*_2_ = 0.1072
*R* indices (all data)	*R*_1_ = 0.0714; *wR*_2_ = 0.1198	*R*_1_ = 0.0783; *wR*_2_ = 0.1493	*R*_1_ = 0.0540; *wR*_2_ =0.1153
Goodness-of-fit on *F*^2^	1.028	1.052	1.030
Min, max e^−^ density (e Å^−3^)	−0.241, 0.199	−0.234, 0.239	−0.239, 0.297

## Supplementary Materials

The following are available online at https://www.mdpi.com/1420-3049/25/3/751/s1: Hydrogen bond data, torsion angles, Hirshfeld surface analysis, PXRD and FTIR spectra. The crystallographic data have been deposited with the CCDC, deposition numbers 1957748-1957750. These data can be obtained free of charge from the Cambridge Crystallographic Data Centre via www.ccdc.cam.ac.uk/data_request/cif.
