# Supplementary material for: Organic Salts of p-Coumaric Acid and Trans-Ferulic Acid with Aminopicolines
Source: Molecules. 2020 Feb 10;25(3):751. doi: 10.3390/molecules25030751 (PMC7037943; doi:10.3390/molecules25030751)
Supplement: Supplementary file 1 [file molecules-25-00751-s001.pdf]

SUPPLEMENTARY DATA

Organic salts of *p*-coumaric acid and *trans*-ferulic acid with aminopicolines

Sosthene Nyomba Kamanda[a] and Ayesha Jacobs*[a]

[a] *Department of Chemistry, Faculty of Applied Sciences, Cape Peninsula University of Technology, PO Box 1906, Cape Town 7535, South Africa. Tel: +27 21 959 3167; E-mail: jacobsa@cput.ac.za.*

HYDROGEN BOND DATA

Table S1. Geometrical data for hydrogen bonds of salt **1**.

	D-H (Å)	H···A (Å)	D···A (Å)	<DHA (Å)	Symmetry operation
N1-H4···O2	0.95	1.71	2.662(2)	173.5	
N2-H9A···O1	0.92	1.98	2.893(3)	171.6	
O3-H1···O1	0.89	1.79	2.671(2)	169.6	x-½, y, -z+$^{3}/_{2}$
N2-H9B···O2	0.82	2.60	3.365(2)	156.3	x+½, -y+½, -z+1

Table S2. Geometrical data for hydrogen bonds of salt **2**.

	D-H (Å)	H···A (Å)	D···A (Å)	<DHA (^{0})	Symmetry operation
N1-H4···O1	0.88	1.87	2.741(3)	169	
N2-H9A···O2	0.93	1.92	2.822(4)	1.65.7	
N3-H14···O4	0.95	1.82	2.753(3)	1.65.5	
N4-H16A···O5	1.00	1.76	2.758(3)	172.3	
N5-H19···O7	0.97	1.80	2.722(2)	1.59.4	
N6-H24A···O8	0.87	2.00	2.845(3)	163.5	
N7-H29···O10	0.97	1.81	2.793(3)	158.6	
N8-H31···O11	0.93	1.87	2.793(3)	167.7	
N2-H9B···O4	0.94	2.05	2.941(3)	156.1	
N4-H16B···O7	0.99	1.90	2.861(3)	162.7	
N6-H24B···O10	0.92	2.06	2.898(3)	151.8	
O3-H1···O8	0.92	1.77	2.668(3)	165.1	-x, y-½, 1-z
O9-H9···O2	0.90	1.72	2.621(3)	175.8	1-x, ½+y, 1-z
N8-H31B···O1	0.96	1.97	2.894(3)	161.0	x, y, 1+z
O6-H10···O5	0.96	1.71	2.666(3)	171.1	-x, ½+y, 1-z
O12-H25···O11	0.89	1.73	2.606(3)	169.1	1-x, y-½, 2-z

Table S3. Geometrical data for hydrogen bonds of salt **3**.

	D-H (Å)	H···A (Å)	D···A (Å)	<DHA (⁰)	Symmetry operation
N1-H1···O2	0.90(3)	1.75 (3)	2.647(3)	172(3)	
N2-H3A···O1	1.05(3)	1.85(3)	2.904(3)	175(2)	
O6-H13···O2	0.97(4)	1.75(4)	2.661(2)	156(4)	
O6-H13···O2	0.95(4)	1.64(4)	2.589(3)	174(3)	
N2-H3B···O4	0.91	2.10(4)	2.964(3)	156(3)	x-½, y+$^{5}/_{2}$, z-½
O5-H9B···O1	0.92(4)	1.84(4)	2.745(3)	169(3)	-x, -y+2, -z
O5-H9A···O6	0.64(4)	2.16(4)	2.789(3)	170(5)	x, y-1, z
C12-H12···O3	0.95	3.28	3.447(3)	92.2	-x, -y+3, -z

TORSION ANGLES

Table S4. Torsion angles of salts **1**, **2** and **3.**

	1	2	3
**Torsion angle**	**(°)**	**(°)**	**(°)**
τ_1(C2-C1-C7-C8)	12.8	14.4	0.2
τ'_1(C17-C16-C22-C23)	-	0.5	-
τ''_1(C32-C31-C37-C38)	-	2.7	-
τ'''_1(C47-C46-C52-C53)	-	4.6	-
τ_2(C7-C8-C9-O1)	10.1	12.8	4.9
τ'_2(C22-C23-C24-O4)	-	26.7	-
τ''_2(C37-C38-C39-O7)	-	9.1	-
τ'''_2(C52-C53-C54-O10)	-	5.1	-

HIRSHFELD SURFACE ANALYSIS

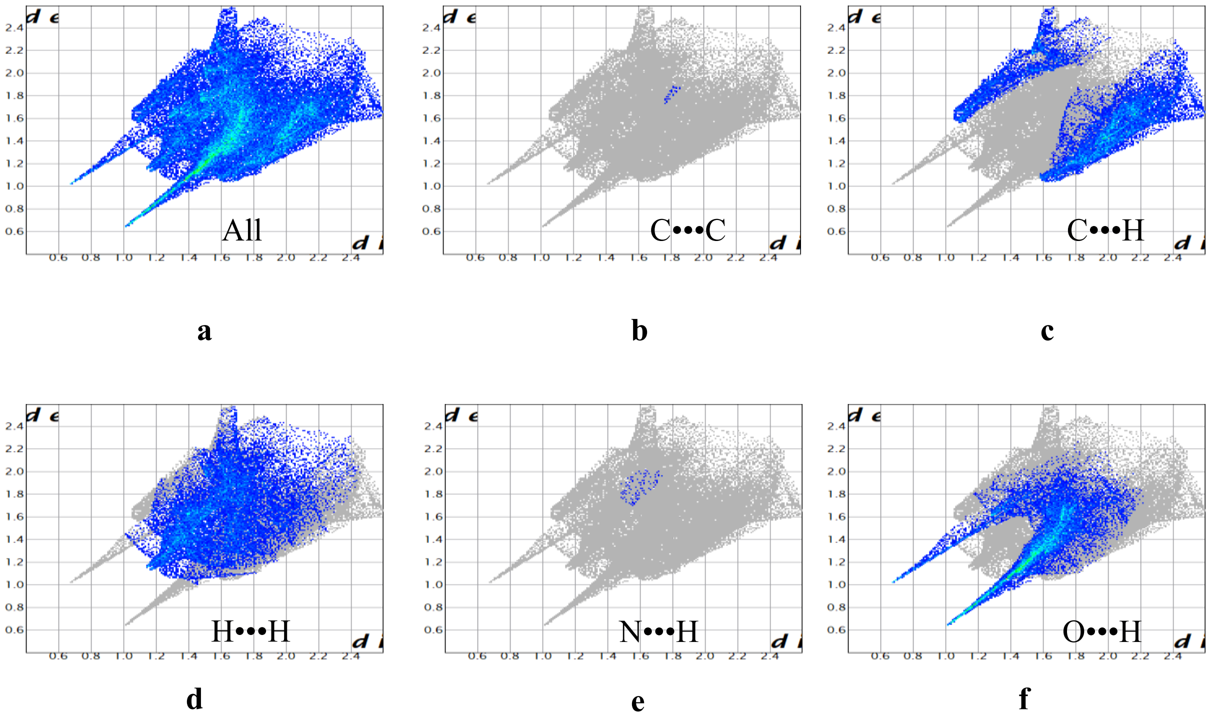

Figure S1. Fingerprint plot of *p*CA in salt **1**.

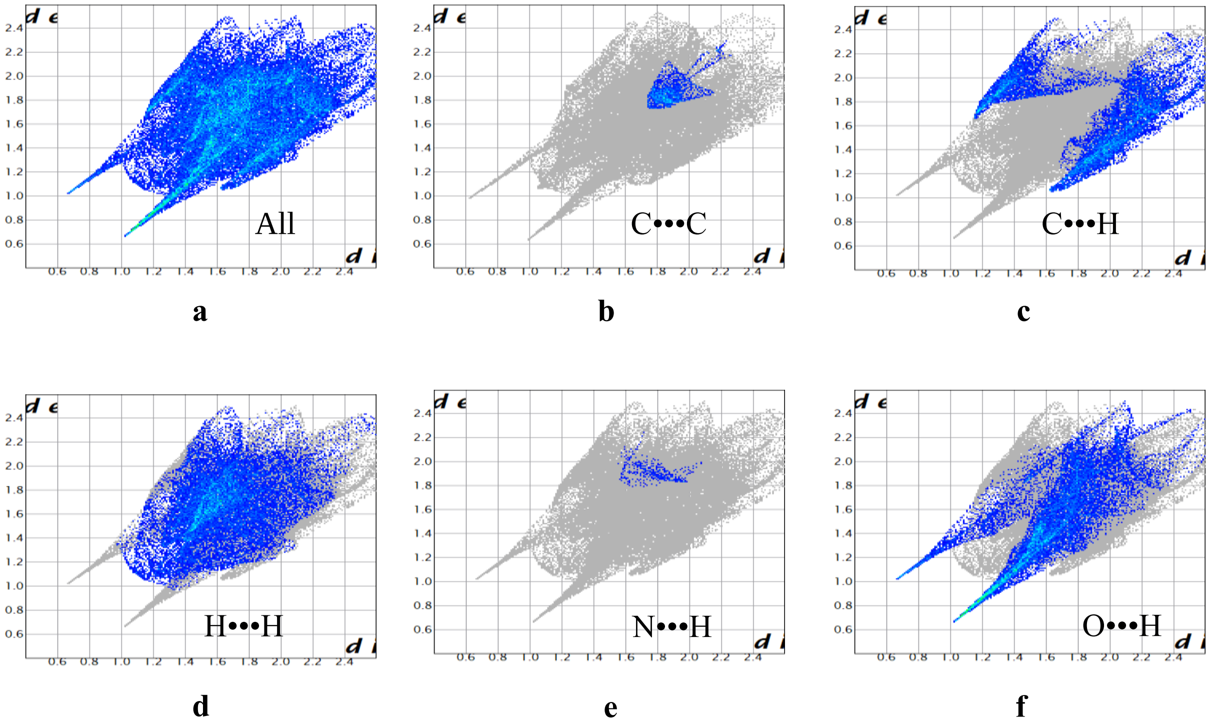

Figure S2 Fingerprint plot of *p*CA in salt **2**.

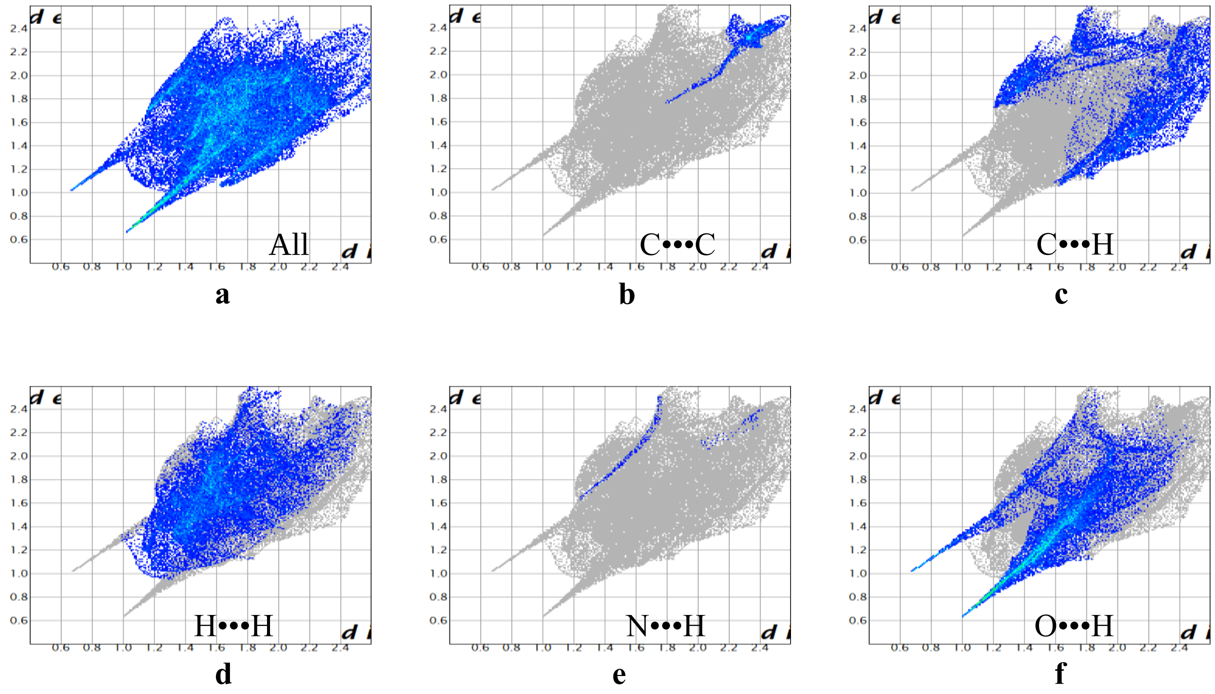

Figure S3. Fingerprint plot of *p*CA′ in salt **2**.

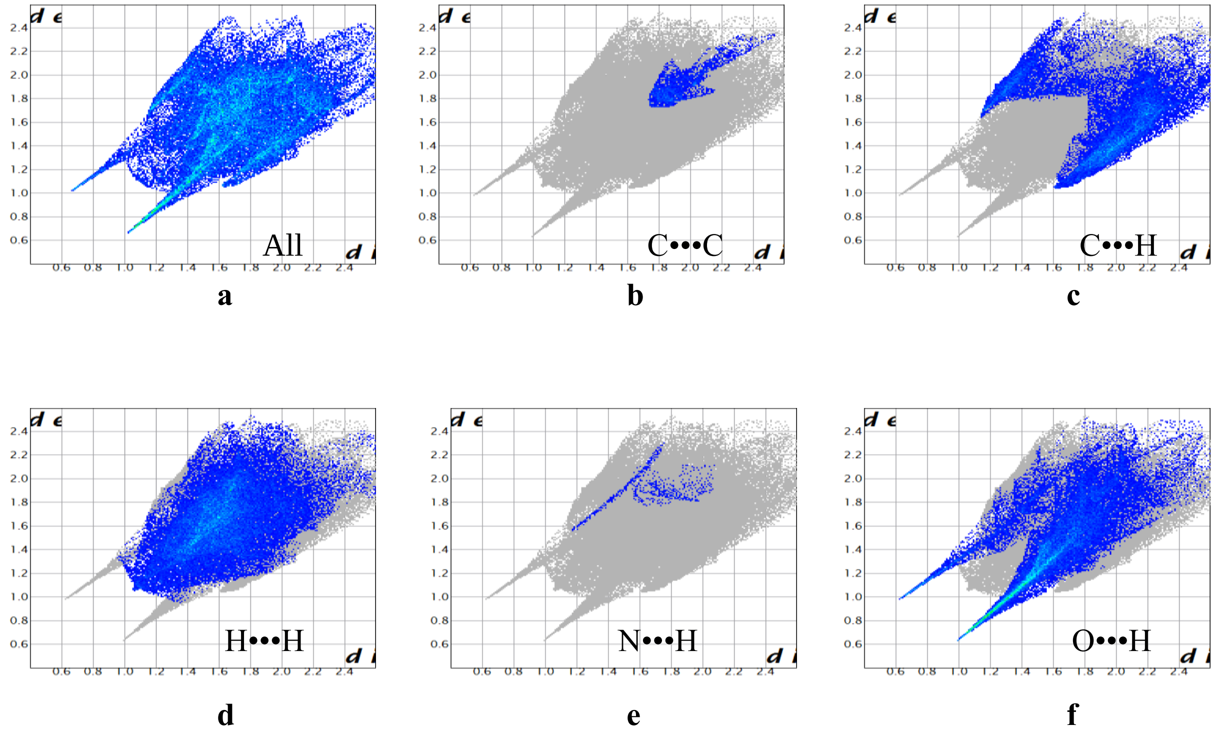

Figure S4. Fingerprint plot of *p*CA″ in salt **2**.

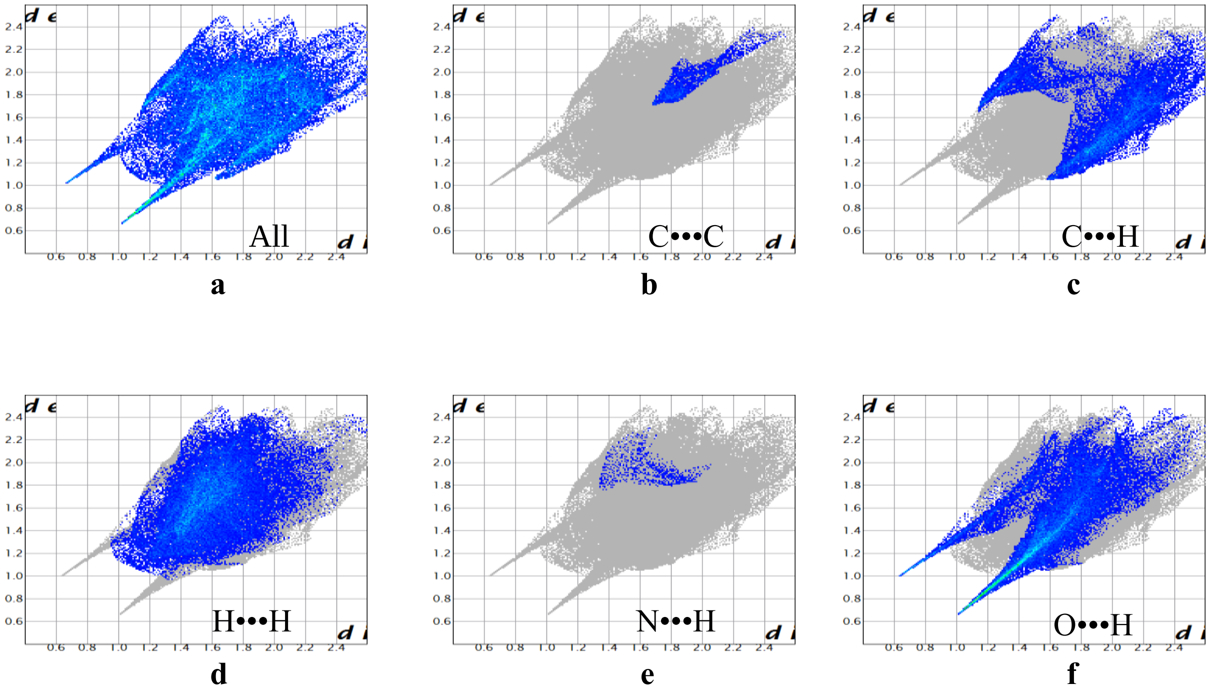

Figure S5. Fingerprint plot of *p*CA‴ in salt **2**.

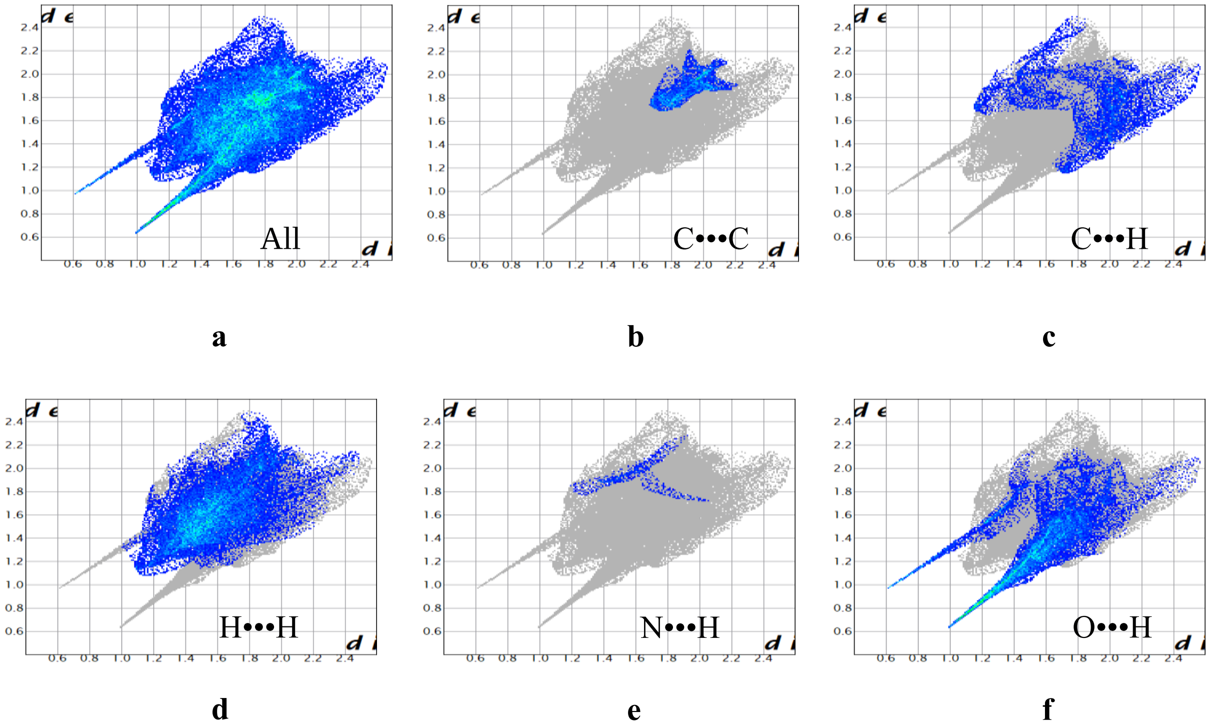

Figure S6. Fingerprint plot of TFA in salt **3**.

Table S5. Quantitative summary of the various interactions of *p*CA and TFA salts.

Salt		C···C (%)	C···H (%)	H···H (%)	N···H (%)	O···H (%)
**1**		0.1	29.6	38.3	0.2	30.1
**2**	*p*CA	1.7	25.2	38.9	0.7	30.8
	*p*CA′	3.4	22.7	38.4	0.5	31.6
	*p*CA′′	2.8	23.3	40	0.7	30.7
	*p*CA′′′	2.5	21.9	39.6	0.8	32.1
**3**		7.5	13.5	45.1	1.3	29.5

GRINDING EXPERIMENTS

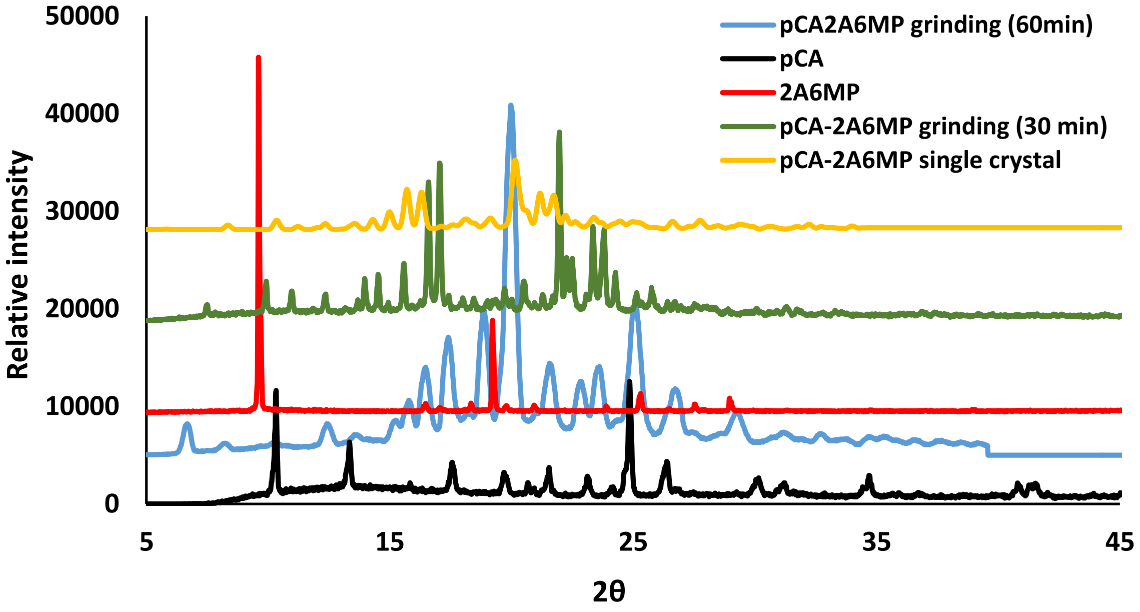

Figure S7. PXRD analyses of *p*CA and 2A6MP grinding (60 min) (blue), *p*CA (black), 2A6MP (red), *p*CA2A6MP grinding (30 min) (green) and the calculated pattern (yellow).

FTIR SPECTRA

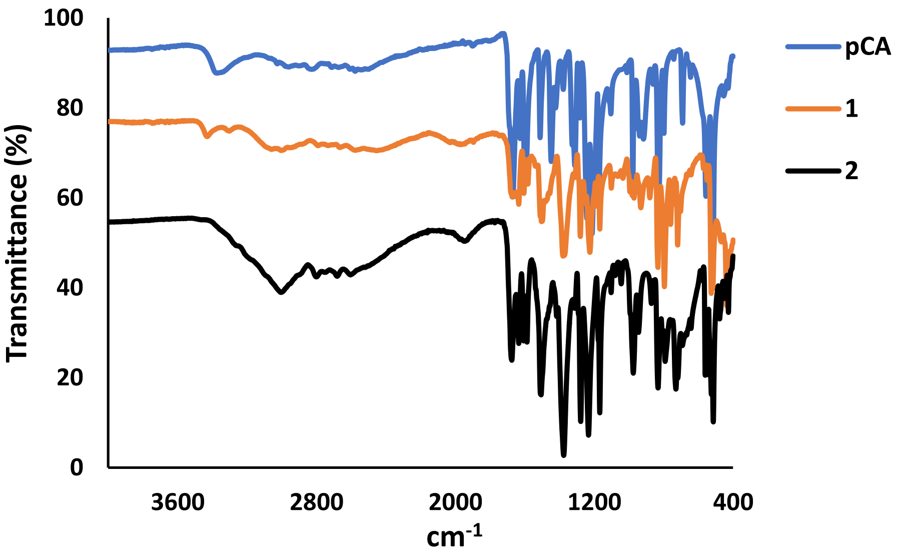

Figure S8. FTIR spectra of *p*CA (blue), salt **1** (orange) and salt **2** (black).

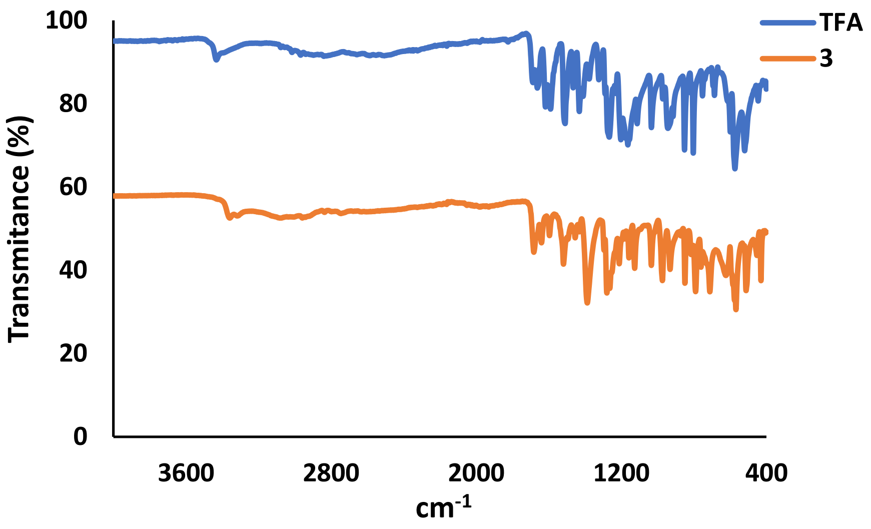

Figure S9. FTIR spectra of TFA (blue) and salt **3** (orange).
